# Crystal structure of benzyl­tri­phenyl­phospho­nium chloride monohydrate

**DOI:** 10.1107/S2056989015009159

**Published:** 2015-05-20

**Authors:** Jimmy Ahmad, Siti Nadiah Abdul Halim, Fiona N.-F. How

**Affiliations:** a Department of Chemistry, Kulliyyah of Science, International Islamic University Malaysia, 25200 Kuantan, Malaysia; b Department of Chemistry, Faculty of Science, Universiti Malaya, Kuala Lumpur 50603, Malaysia

**Keywords:** crystal structure, benzyl­tri­phenyl­phospho­nium, chloride, hydrogen bonding, C—H⋯ π inter­actions

## Abstract

The title compound, Ph_3_(PhCH_2_)P^+^·Cl^−^·H_2_O, was obtained unintentionally as the product of an attempted synthesis of a silver di­thio­carbamate complex using benzyl­tri­phenyl­phospho­nium as the counter-ion. The asymmetric unit consists of a phospho­nium cation and a chloride anion, and a water mol­ecule of crystallization. In the crystal, the chloride ion is linked to the water mol­ecule by an O—H⋯Cl hydrogen bond. The three units are further linked *via* C—H⋯Cl and C—H⋯O hydrogen bonds and C—H⋯ π inter­actions, forming a three-dimensional structure.

## Related literature   

For some structures containing the Ph_3_(PhCH_2_)P^+^ cation, see: Li & He (2011 ▸); Fischer & Wiebelhaus (1997 ▸); Skapski & Stephens (1974 ▸).
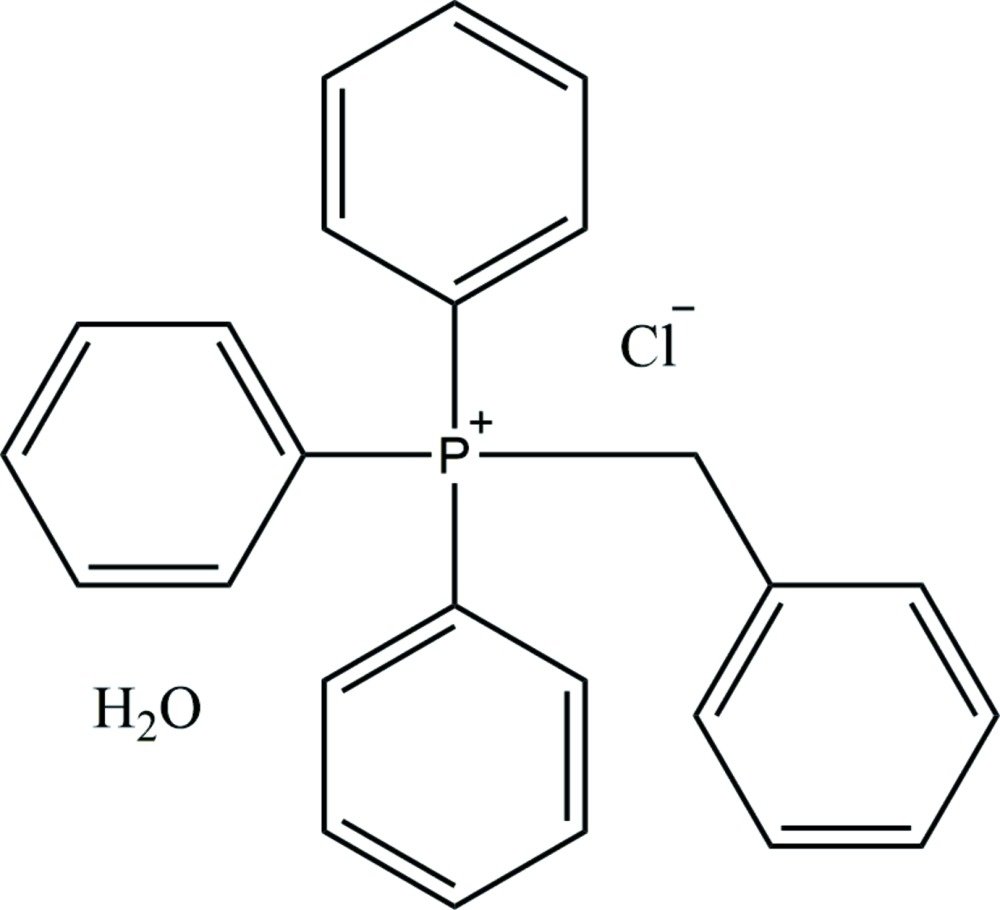


## Experimental   

### Crystal data  

C_25_H_22_P^+^·Cl^−^·H_2_O
*M* *_r_* = 406.86
Monoclinic, 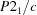

*a* = 9.7368 (8) Å
*b* = 19.7474 (17) Å
*c* = 11.4170 (9) Å
β = 109.728 (9)°
*V* = 2066.4 (3) Å^3^
*Z* = 4
Mo *K*α radiation
μ = 0.28 mm^−1^
*T* = 100 K
0.30 × 0.25 × 0.20 mm

### Data collection  

Agilent SuperNova (Dual, Cu at zero, Atlas) diffractometer
Absorption correction: multi-scan (*CrysAlis PRO*; Agilent 2013 ▸) *T*_min_ = 0.813, *T*_max_ = 1.000
12625 measured reflections
5434 independent reflections
3901 reflections with *I* > 2σ(*I*)
*R*_int_ = 0.067

### Refinement  

*R*[*F* ^2^ > 2σ(*F* ^2^)] = 0.067
*wR*(*F* ^2^) = 0.194
*S* = 1.07
5434 reflections
256 parameters
H-atom parameters constrained
Δρ_max_ = 0.90 e Å^−3^
Δρ_min_ = −0.72 e Å^−3^

### 

Data collection: *CrysAlis PRO* (Agilent, 2013 ▸); cell refinement: *CrysAlis PRO*; data reduction: *CrysAlis PRO*; program(s) used to solve structure: *OLEX2.solve* (Bourhis *et al.*, 2015 ▸); program(s) used to refine structure: *SHELXL2013* (Sheldrick, 2015 ▸); molecular graphics: *OLEX2* (Dolomanov *et al.*, 2009 ▸); software used to prepare material for publication: *publCIF* (Westrip, 2010 ▸).

## Supplementary Material

Crystal structure: contains datablock(s) I. DOI: 10.1107/S2056989015009159/su5134sup1.cif

Structure factors: contains datablock(s) I. DOI: 10.1107/S2056989015009159/su5134Isup2.hkl

Supporting information file. DOI: 10.1107/S2056989015009159/su5134Isup3.cml

**Fig. 1.**
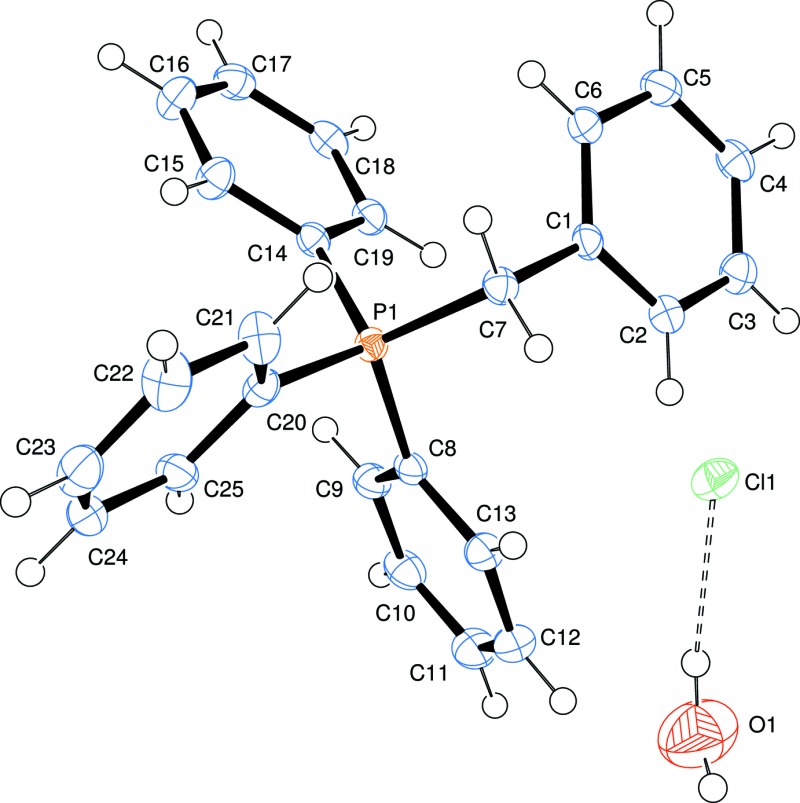
The asymmetric unit of the title compound, with atom labelling. Displacement ellipsoids are drawn at the 50% probability level. Dotted line denotes the O—H···Cl hydrogen bond (see Table 1 for details).

. DOI: 10.1107/S2056989015009159/su5134fig1.tif
The asymmetric unit of the title compound, with atom labelling. Displacement ellipsoids are drawn at the 50% probability level. Dotted line denotes the O—H⋯Cl hydrogen bond (see Table 1 for details).

CCDC reference: 1400555

Additional supporting information:  crystallographic information; 3D view; checkCIF report

## Figures and Tables

**Table 1:** Hydrogen-bond geometry (, ) *Cg*2 and *Cg*4 are the centroids of rings C8-C13 and C20-C25, respectively.

*D*H*A*	*D*H	H*A*	*D* *A*	*D*H*A*
O1H1*B*Cl1^i^	0.85	2.27	3.114(3)	170
C7H7*A*Cl1	0.97	2.57	3.511(3)	162
C7H7*B*Cl1^ii^	0.97	2.60	3.528(2)	160
C12H12O1^iii^	0.93	2.47	3.207(5)	136
C17H17Cl1^iv^	0.93	2.81	3.562(3)	139
C3H3*Cg*4^v^	0.93	2.83	3.584(3)	139
C18H18*Cg*2^vi^	0.93	2.98	3.720(3)	137

Symmetry codes: (i) 
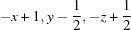
; (ii) 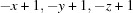; (iii) 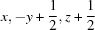; (iv) 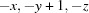; (v) 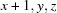; (vi) 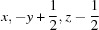.

## supplementary crystallographic information

### S1. Synthesis and crystallization

The title compound was obtained unintentionally as the product of an attempted synthesis of silver complex of di­thio­carbamate using benzyl­tri­phenyl­phospho­nium as the counter ion. Colourless crystals were obtained upon slow evaporation of the methano­lic solution at room temperature.

### S2. Refinement

Crystal data, data collection and structure refinement details are summarized in Table 2. The H atoms of the water molecule were located in a Fourier difference map. The water molecule was then refined as a rigid group with U_iso_(H) = 1.5U_eq_(O). The C-bound H atoms were included in calculated positions and treated as riding atoms: C—H = 0.93 Å with U_iso_(H) = 1.2U_eq_(C).

### S3. Results and discussion

The asymmetric unit of the title compound, shown in Fig. 1, consists of one independent cation, one independent anion and a hydrated water molecule. The central phosphine atom coordinates with the ligands in a slightly distorted tetra­hedral environment. The C—P—C bond angles vary from 108.56 (12) to 110.51 (11) °, deviating slightly from the ideal tetra­hedral angle of 109.5 °. The P–C bond distances, that vary from 1.792 (2) to 1.800 (3) Å, are comparable to values found for related compounds containing the Ph_3_(PhCH_2_)P^+^ cation (Li & He, 2011; Fischer & Wiebelhaus, 1997; Skapski & Stephens, 1974).

In the crystal, the chloride ion is linked to the water molecule by an O—H···Cl hydrogen bond (Table 1 and Fig. 1). The three units are further linked *via* C—H···Cl and C—H···O hydrogen bonds and C—H··· π inter­actions (Table 1) forming a three-dimensional structure.

### S4. Experimental

The title compound was obtained unintentionally as the product of an attempted synthesis of silver complex of dithiocarbamate using benzyltriphenylphosphonium as the counter ion. The colourless crystal was obtained upon slow evaporation of the methanolic solution at room temperature.

### Crystal data

**Table:**

C_25_H_22_P^+^·Cl^−^·H_2_O	*F*(000) = 856
*M**_r_* = 406.86	*D*_x_ = 1.308 Mg m^−^^3^
Monoclinic, *P*2_1_/*c*	Mo *K*α radiation, λ = 0.71073 Å
*a* = 9.7368 (8) Å	Cell parameters from 2649 reflections
*b* = 19.7474 (17) Å	θ = 3.6–30.1°
*c* = 11.4170 (9) Å	µ = 0.28 mm^−^^1^
β = 109.728 (9)°	*T* = 100 K
*V* = 2066.4 (3) Å^3^	Block, colourless
*Z* = 4	0.30 × 0.25 × 0.20 mm

### Data collection

**Table:**

Agilent SuperNova (Dual, Cu at zero, Atlas) diffractometer	5434 independent reflections
Radiation source: SuperNova (Mo) X-ray Source	3901 reflections with *I* > 2σ(*I*)
Mirror monochromator	*R*_int_ = 0.067
Detector resolution: 10.4041 pixels mm^-1^	θ_max_ = 30.3°, θ_min_ = 3.0°
ω scans	*h* = −13→13
Absorption correction: multi-scan (*CrysAlis PRO*; Agilent 2013)	*k* = −26→18
*T*_min_ = 0.813, *T*_max_ = 1.000	*l* = −16→15
12625 measured reflections	

### Refinement

**Table:**

Refinement on *F*^2^	Primary atom site location: iterative
Least-squares matrix: full	Secondary atom site location: difference Fourier map
*R*[*F*^2^ > 2σ(*F*^2^)] = 0.067	Hydrogen site location: inferred from neighbouring sites
*wR*(*F*^2^) = 0.194	H-atom parameters constrained
*S* = 1.07	*w* = 1/[σ^2^(*F*_o_^2^) + (0.0844*P*)^2^ + 1.2017*P*] where *P* = (*F*_o_^2^ + 2*F*_c_^2^)/3
5434 reflections	(Δ/σ)_max_ < 0.001
256 parameters	Δρ_max_ = 0.90 e Å^−^^3^
0 restraints	Δρ_min_ = −0.72 e Å^−^^3^

### Special details

**Table:**

Geometry. All e.s.d.'s (except the e.s.d. in the dihedral angle between two l.s. planes) are estimated using the full covariance matrix. The cell e.s.d.'s are taken into account individually in the estimation of e.s.d.'s in distances, angles and torsion angles; correlations between e.s.d.'s in cell parameters are only used when they are defined by crystal symmetry. An approximate (isotropic) treatment of cell e.s.d.'s is used for estimating e.s.d.'s involving l.s. planes.
Refinement. Refinement of *F*^2^ against ALL reflections. The weighted *R*-factor *wR* and goodness of fit *S* are based on *F*^2^, conventional *R*-factors *R* are based on *F*, with *F* set to zero for negative *F*^2^. The threshold expression of *F*^2^ > σ(*F*^2^) is used only for calculating *R*-factors(gt) *etc*. and is not relevant to the choice of reflections for refinement. *R*-factors based on *F*^2^ are statistically about twice as large as those based on *F*, and *R*- factors based on ALL data will be even larger.Carbon-bound H-atoms were placed in calculated positions (C–H 0.93–0.97 Å) and were included in the refinement in the riding model approximation with *U*_iso_(H) = 1.2*U*_eq_(C). H atoms in water molecule (O–H 0.85 Å) were refined using a riding model with *U*_iso_(H) = 1.5*U*_eq_(O).

### Fractional atomic coordinates and isotropic or equivalent isotropic displacement parameters (Å^2^)

**Table:**

	*x*	*y*	*z*	*U*_iso_*/*U*_eq_	
Cl1	0.25025 (7)	0.57674 (4)	0.31223 (6)	0.02397 (19)	
P1	0.34083 (7)	0.36232 (3)	0.34126 (5)	0.01553 (18)	
C18	0.2330 (3)	0.37275 (14)	−0.0334 (2)	0.0213 (5)	
H18	0.2775	0.3645	−0.0923	0.026*	
C7	0.4679 (3)	0.43096 (13)	0.3966 (2)	0.0178 (5)	
H7A	0.4141	0.4727	0.3933	0.021*	
H7B	0.5262	0.4227	0.4829	0.021*	
C8	0.4377 (3)	0.28343 (13)	0.3638 (2)	0.0173 (5)	
C24	0.0575 (3)	0.29764 (17)	0.5078 (2)	0.0270 (6)	
H24	0.0103	0.2574	0.5131	0.032*	
C3	0.7862 (3)	0.41102 (16)	0.2782 (3)	0.0260 (6)	
H3	0.8712	0.3856	0.2960	0.031*	
C13	0.5493 (3)	0.27260 (14)	0.4777 (2)	0.0219 (6)	
H13	0.5710	0.3055	0.5396	0.026*	
C20	0.2189 (3)	0.36118 (14)	0.4295 (2)	0.0187 (5)	
C5	0.6222 (3)	0.49415 (15)	0.1536 (2)	0.0239 (6)	
H5	0.5977	0.5252	0.0885	0.029*	
C6	0.5303 (3)	0.48535 (14)	0.2233 (2)	0.0205 (5)	
H6	0.4441	0.5100	0.2042	0.025*	
C4	0.7491 (3)	0.45721 (15)	0.1805 (2)	0.0246 (6)	
H4	0.8098	0.4631	0.1334	0.030*	
C15	0.0985 (3)	0.39724 (15)	0.1413 (2)	0.0226 (6)	
H15	0.0535	0.4058	0.1998	0.027*	
C14	0.2409 (3)	0.37351 (13)	0.1790 (2)	0.0163 (5)	
C19	0.3092 (3)	0.36202 (13)	0.0909 (2)	0.0195 (5)	
H19	0.4054	0.3472	0.1161	0.023*	
C1	0.5683 (3)	0.43952 (13)	0.3214 (2)	0.0174 (5)	
C10	0.4851 (3)	0.17419 (15)	0.2931 (3)	0.0257 (6)	
H10	0.4640	0.1412	0.2313	0.031*	
C11	0.5955 (3)	0.16351 (16)	0.4041 (3)	0.0290 (6)	
H11	0.6491	0.1236	0.4171	0.035*	
C16	0.0233 (3)	0.40826 (16)	0.0154 (2)	0.0263 (6)	
H16	−0.0722	0.4241	−0.0103	0.032*	
C17	0.0900 (3)	0.39582 (15)	−0.0711 (2)	0.0240 (6)	
H17	0.0391	0.4029	−0.1552	0.029*	
C9	0.4051 (3)	0.23357 (14)	0.2723 (2)	0.0215 (5)	
H9	0.3297	0.2402	0.1973	0.026*	
C22	0.1048 (3)	0.41462 (18)	0.5617 (3)	0.0313 (7)	
H22	0.0892	0.4528	0.6031	0.038*	
C25	0.1486 (3)	0.30063 (15)	0.4365 (2)	0.0223 (6)	
H25	0.1624	0.2625	0.3939	0.027*	
C12	0.6270 (3)	0.21230 (16)	0.4971 (3)	0.0282 (6)	
H12	0.7006	0.2045	0.5727	0.034*	
C21	0.1962 (3)	0.41847 (16)	0.4911 (2)	0.0255 (6)	
H21	0.2418	0.4591	0.4851	0.031*	
C2	0.6968 (3)	0.40286 (15)	0.3491 (2)	0.0224 (6)	
H2	0.7229	0.3727	0.4155	0.027*	
C23	0.0372 (3)	0.35462 (17)	0.5707 (2)	0.0294 (7)	
H23	−0.0224	0.3523	0.6193	0.035*	
O1	0.8349 (4)	0.22715 (17)	0.2572 (3)	0.0728 (10)	
H1A	0.8551	0.2282	0.3357	0.109*	
H1B	0.8042	0.1879	0.2301	0.109*	

### Atomic displacement parameters (Å^2^)

**Table:**

	*U*^11^	*U*^22^	*U*^33^	*U*^12^	*U*^13^	*U*^23^
Cl1	0.0225 (3)	0.0253 (4)	0.0235 (3)	0.0063 (3)	0.0070 (2)	0.0027 (2)
P1	0.0149 (3)	0.0154 (3)	0.0171 (3)	0.0005 (2)	0.0063 (2)	−0.0006 (2)
C18	0.0283 (14)	0.0186 (13)	0.0210 (11)	−0.0047 (11)	0.0135 (10)	−0.0034 (10)
C7	0.0177 (12)	0.0158 (13)	0.0201 (11)	−0.0006 (10)	0.0066 (9)	−0.0017 (9)
C8	0.0164 (11)	0.0161 (13)	0.0228 (11)	0.0015 (10)	0.0112 (9)	0.0031 (9)
C24	0.0219 (13)	0.0311 (16)	0.0307 (14)	0.0016 (13)	0.0124 (11)	0.0099 (12)
C3	0.0193 (13)	0.0267 (16)	0.0328 (14)	0.0012 (12)	0.0098 (11)	0.0016 (11)
C13	0.0210 (13)	0.0214 (14)	0.0240 (12)	0.0010 (11)	0.0083 (10)	0.0020 (10)
C20	0.0163 (12)	0.0243 (14)	0.0147 (11)	0.0036 (11)	0.0043 (9)	0.0014 (9)
C5	0.0281 (14)	0.0210 (14)	0.0211 (12)	−0.0041 (12)	0.0063 (10)	0.0025 (10)
C6	0.0183 (12)	0.0174 (13)	0.0245 (12)	−0.0019 (11)	0.0054 (9)	−0.0005 (10)
C4	0.0250 (14)	0.0258 (16)	0.0278 (13)	−0.0045 (12)	0.0152 (11)	−0.0001 (11)
C15	0.0200 (13)	0.0278 (15)	0.0206 (11)	0.0038 (12)	0.0079 (10)	−0.0007 (10)
C14	0.0177 (12)	0.0157 (12)	0.0157 (10)	−0.0024 (10)	0.0059 (9)	−0.0013 (9)
C19	0.0194 (12)	0.0171 (13)	0.0242 (12)	−0.0001 (10)	0.0102 (10)	−0.0008 (10)
C1	0.0147 (11)	0.0151 (12)	0.0217 (11)	−0.0028 (10)	0.0053 (9)	−0.0020 (9)
C10	0.0310 (15)	0.0169 (14)	0.0336 (14)	−0.0013 (12)	0.0165 (12)	−0.0020 (11)
C11	0.0287 (15)	0.0198 (15)	0.0414 (15)	0.0066 (13)	0.0157 (12)	0.0032 (12)
C16	0.0208 (13)	0.0320 (17)	0.0242 (13)	0.0045 (12)	0.0053 (10)	0.0010 (11)
C17	0.0291 (14)	0.0220 (14)	0.0187 (11)	−0.0037 (12)	0.0053 (10)	0.0013 (10)
C9	0.0232 (13)	0.0172 (13)	0.0253 (12)	0.0002 (11)	0.0097 (10)	0.0007 (10)
C22	0.0288 (15)	0.042 (2)	0.0265 (13)	0.0001 (14)	0.0136 (12)	−0.0091 (12)
C25	0.0245 (13)	0.0207 (14)	0.0247 (12)	0.0017 (11)	0.0124 (10)	0.0041 (10)
C12	0.0243 (14)	0.0256 (16)	0.0332 (14)	0.0043 (12)	0.0076 (11)	0.0080 (12)
C21	0.0232 (13)	0.0265 (16)	0.0289 (13)	−0.0035 (12)	0.0115 (11)	−0.0078 (11)
C2	0.0191 (12)	0.0230 (14)	0.0243 (12)	−0.0028 (11)	0.0063 (10)	0.0028 (10)
C23	0.0230 (13)	0.047 (2)	0.0214 (12)	0.0061 (14)	0.0116 (11)	0.0058 (12)
O1	0.080 (2)	0.053 (2)	0.0685 (19)	−0.0064 (18)	0.0032 (18)	−0.0002 (15)

### Geometric parameters (Å, º)

**Table:**

P1—C7	1.800 (3)	C4—H4	0.9300
P1—C8	1.794 (3)	C15—H15	0.9300
P1—C20	1.798 (3)	C15—C14	1.387 (4)
P1—C14	1.792 (2)	C15—C16	1.393 (4)
C18—H18	0.9300	C14—C19	1.398 (3)
C18—C19	1.378 (3)	C19—H19	0.9300
C18—C17	1.388 (4)	C1—C2	1.386 (4)
C7—H7A	0.9700	C10—H10	0.9300
C7—H7B	0.9700	C10—C11	1.374 (4)
C7—C1	1.512 (4)	C10—C9	1.383 (4)
C8—C13	1.401 (4)	C11—H11	0.9300
C8—C9	1.392 (4)	C11—C12	1.389 (4)
C24—H24	0.9300	C16—H16	0.9300
C24—C25	1.392 (4)	C16—C17	1.374 (4)
C24—C23	1.385 (4)	C17—H17	0.9300
C3—H3	0.9300	C9—H9	0.9300
C3—C4	1.391 (4)	C22—H22	0.9300
C3—C2	1.384 (4)	C22—C21	1.390 (4)
C13—H13	0.9300	C22—C23	1.376 (5)
C13—C12	1.388 (4)	C25—H25	0.9300
C20—C25	1.394 (4)	C12—H12	0.9300
C20—C21	1.388 (4)	C21—H21	0.9300
C5—H5	0.9300	C2—H2	0.9300
C5—C6	1.395 (4)	C23—H23	0.9300
C5—C4	1.377 (4)	O1—H1A	0.8504
C6—H6	0.9300	O1—H1B	0.8496
C6—C1	1.390 (4)		
			
C8—P1—C7	109.74 (12)	C15—C14—C19	120.0 (2)
C8—P1—C20	108.86 (12)	C19—C14—P1	119.98 (19)
C20—P1—C7	108.56 (12)	C18—C19—C14	119.6 (2)
C14—P1—C7	109.82 (12)	C18—C19—H19	120.2
C14—P1—C8	109.33 (12)	C14—C19—H19	120.2
C14—P1—C20	110.51 (11)	C6—C1—C7	118.9 (2)
C19—C18—H18	119.8	C2—C1—C7	121.2 (2)
C19—C18—C17	120.3 (2)	C2—C1—C6	119.9 (2)
C17—C18—H18	119.8	C11—C10—H10	119.7
P1—C7—H7A	109.1	C11—C10—C9	120.5 (3)
P1—C7—H7B	109.1	C9—C10—H10	119.7
H7A—C7—H7B	107.8	C10—C11—H11	120.0
C1—C7—P1	112.58 (17)	C10—C11—C12	120.1 (3)
C1—C7—H7A	109.1	C12—C11—H11	120.0
C1—C7—H7B	109.1	C15—C16—H16	119.9
C13—C8—P1	118.1 (2)	C17—C16—C15	120.2 (3)
C9—C8—P1	122.10 (19)	C17—C16—H16	119.9
C9—C8—C13	119.8 (2)	C18—C17—H17	119.9
C25—C24—H24	120.0	C16—C17—C18	120.2 (2)
C23—C24—H24	120.0	C16—C17—H17	119.9
C23—C24—C25	119.9 (3)	C8—C9—H9	120.0
C4—C3—H3	120.0	C10—C9—C8	119.9 (2)
C2—C3—H3	120.0	C10—C9—H9	120.0
C2—C3—C4	120.1 (3)	C21—C22—H22	119.8
C8—C13—H13	120.4	C23—C22—H22	119.8
C12—C13—C8	119.3 (3)	C23—C22—C21	120.4 (3)
C12—C13—H13	120.4	C24—C25—C20	119.5 (3)
C25—C20—P1	118.2 (2)	C24—C25—H25	120.3
C21—C20—P1	121.4 (2)	C20—C25—H25	120.3
C21—C20—C25	120.3 (2)	C13—C12—C11	120.3 (3)
C6—C5—H5	119.8	C13—C12—H12	119.8
C4—C5—H5	119.8	C11—C12—H12	119.8
C4—C5—C6	120.5 (2)	C20—C21—C22	119.4 (3)
C5—C6—H6	120.2	C20—C21—H21	120.3
C1—C6—C5	119.6 (2)	C22—C21—H21	120.3
C1—C6—H6	120.2	C3—C2—C1	120.3 (2)
C3—C4—H4	120.1	C3—C2—H2	119.9
C5—C4—C3	119.8 (3)	C1—C2—H2	119.9
C5—C4—H4	120.1	C24—C23—H23	119.8
C14—C15—H15	120.2	C22—C23—C24	120.4 (3)
C14—C15—C16	119.7 (2)	C22—C23—H23	119.8
C16—C15—H15	120.2	H1A—O1—H1B	109.5
C15—C14—P1	119.93 (19)		
			
P1—C7—C1—C6	94.1 (3)	C6—C5—C4—C3	−0.3 (4)
P1—C7—C1—C2	−86.2 (3)	C6—C1—C2—C3	−0.9 (4)
P1—C8—C13—C12	−179.3 (2)	C4—C3—C2—C1	1.4 (4)
P1—C8—C9—C10	178.5 (2)	C4—C5—C6—C1	0.8 (4)
P1—C20—C25—C24	177.77 (19)	C15—C14—C19—C18	−1.6 (4)
P1—C20—C21—C22	−177.8 (2)	C15—C16—C17—C18	−0.5 (5)
P1—C14—C19—C18	−178.0 (2)	C14—P1—C7—C1	−54.7 (2)
C7—P1—C8—C13	44.1 (2)	C14—P1—C8—C13	164.6 (2)
C7—P1—C8—C9	−135.8 (2)	C14—P1—C8—C9	−15.3 (3)
C7—P1—C20—C25	−156.91 (19)	C14—P1—C20—C25	82.6 (2)
C7—P1—C20—C21	22.1 (2)	C14—P1—C20—C21	−98.4 (2)
C7—P1—C14—C15	−102.9 (2)	C14—C15—C16—C17	−0.1 (5)
C7—P1—C14—C19	73.5 (2)	C19—C18—C17—C16	0.1 (4)
C7—C1—C2—C3	179.4 (2)	C10—C11—C12—C13	−1.2 (5)
C8—P1—C7—C1	65.5 (2)	C11—C10—C9—C8	0.9 (4)
C8—P1—C20—C25	−37.5 (2)	C16—C15—C14—P1	177.6 (2)
C8—P1—C20—C21	141.5 (2)	C16—C15—C14—C19	1.2 (4)
C8—P1—C14—C15	136.6 (2)	C17—C18—C19—C14	1.0 (4)
C8—P1—C14—C19	−47.0 (2)	C9—C8—C13—C12	0.7 (4)
C8—C13—C12—C11	0.6 (4)	C9—C10—C11—C12	0.4 (4)
C13—C8—C9—C10	−1.4 (4)	C25—C24—C23—C22	1.1 (4)
C20—P1—C7—C1	−175.62 (17)	C25—C20—C21—C22	1.2 (4)
C20—P1—C8—C13	−74.5 (2)	C21—C20—C25—C24	−1.3 (4)
C20—P1—C8—C9	105.5 (2)	C21—C22—C23—C24	−1.1 (4)
C20—P1—C14—C15	16.8 (3)	C2—C3—C4—C5	−0.8 (4)
C20—P1—C14—C19	−166.8 (2)	C23—C24—C25—C20	0.1 (4)
C5—C6—C1—C7	179.6 (2)	C23—C22—C21—C20	0.0 (4)
C5—C6—C1—C2	−0.1 (4)		

### Hydrogen-bond geometry (Å, º)

Cg2 and Cg4 are the centroids of rings C8-C13 and C20-C25, respectively.

**Table:**

*D*—H···*A*	*D*—H	H···*A*	*D*···*A*	*D*—H···*A*
O1—H1*B*···Cl1^i^	0.85	2.27	3.114 (3)	170
C7—H7*A*···Cl1	0.97	2.57	3.511 (3)	162
C7—H7*B*···Cl1^ii^	0.97	2.60	3.528 (2)	160
C12—H12···O1^iii^	0.93	2.47	3.207 (5)	136
C17—H17···Cl1^iv^	0.93	2.81	3.562 (3)	139
C3—H3···*Cg*4^v^	0.93	2.83	3.584 (3)	139
C18—H18···*Cg*2^vi^	0.93	2.98	3.720 (3)	137

Symmetry codes: (i) −*x*+1, *y*−1/2, −*z*+1/2; (ii) −*x*+1, −*y*+1, −*z*+1; (iii) *x*, −*y*+1/2, *z*+1/2; (iv) −*x*, −*y*+1, −*z*; (v) *x*+1, *y*, *z*; (vi) *x*, −*y*+1/2, *z*−1/2.
