# Supplementary figures and images for: Crystal structure of benzyl­tri­phenyl­phospho­nium chloride monohydrate
Source: Acta Crystallogr E Crystallogr Commun. 2015 May 20;71(Pt 6):o410–1. doi: 10.1107/S2056989015009159 (PMC4459357; doi:10.1107/S2056989015009159)

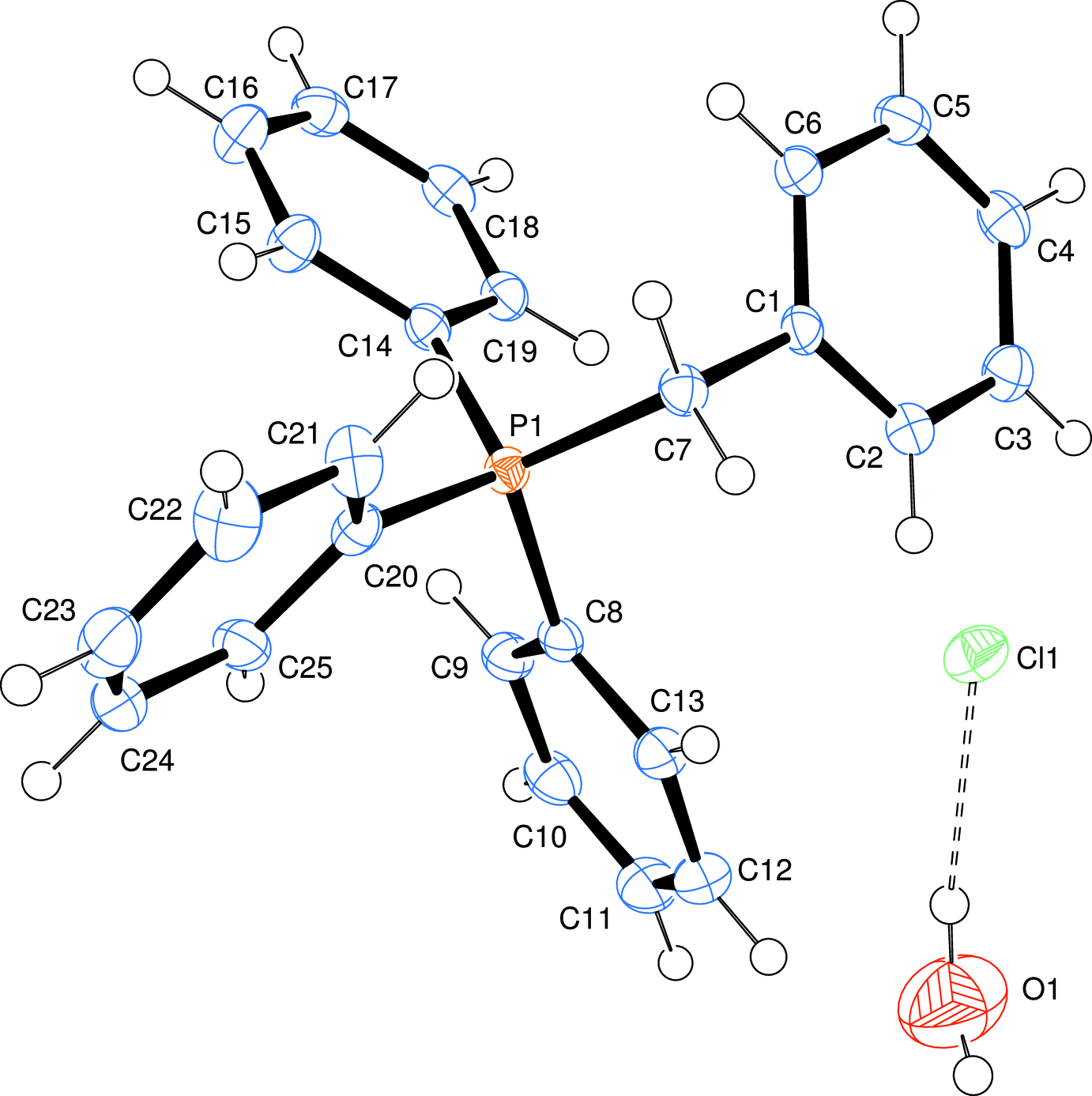

Supplement: Supplementary file 4 [file e-71-0o410-fig1.tif]
